# Fruits in Summer

**Published:** 1856-07

**Authors:** 


### FRUITS IN SUMMER.

By an arrangement of Providence, as beautiful as it is benign, the fruits of the earth are ripening during the whole summer. From the delightful strawberry on the opening of spring, to the luscious peach of the fall, there is a constant succession of delightful aliments; made delightful by that Power, whose loving kindness is in all his works, in order to stimulate us to their highest cultivation, connecting with their use also, the most health giving influences; and with the rich profuseness of a well attended fruitery, it is one of the most unaccountable things in nature, that so little attention is paid, comparatively speaking, to this branch of farming.

It is a beautiful fact, that while the warmth and exposures of summer tend to biliousness and fevers, the free use of fruits and berries counteract that tendency. Artificial acids are found to promote the separation of the bile from the blood, with great mildness and certainty; this led to the supposition, that the natural acids, as contained in fruits and berries, might be as available, and being more palatable, would necessarily be preferred. Experiment has verified the theory, and within a very late period, Allopathic writers have suggested the use of fresh, ripe, perfect, raw fruits, as a reliable remedy in the diarrhœas of summer.

How strongly the appetite yearns for a pickle, when nothing else could be relished, is in the experience of most of us. It is the instinct of nature, pointing to a cure. The want of a natural appetite, is the result of the bile not being separated from the blood, and if not remedied, fever is inevitable, from the slightest grades, to that of bilious, congestive and yellow. "Fruits are cooling," is a bye-word, the truth of which has forced itself on the commonest observers. But why they are so, they had not the time, opportunity or inclination to inquire into. The reason is, the acid of the fruit stimulates the liver to greater activity in separating the bile from the blood, which is its proper work, the result of which is, the bowels become free, the pores of the skin are open. Under such circumstances, fever and want of appetite, are impossible.

HOW TO USE FRUITS.

To derive from the employment of fruits and berries all that healthful and nutritive effect which belongs to their nature, we should

*First*—Use fruits that are ripe, fresh, perfect, raw.

*Second*—They should be used in their natural state, without sugar, cream, milk or any other item of food or drink.

*Third*—Fruits have their best effect when used in the early part of the day, hence we do not advise their employment at a later hour than the middle of the afternoon; not that, if perfect and ripe, they may not be eaten largely by themselves, within two hours of bed time, with advantage, but if the sourness of decay should happen to taint them or any liquor should inadvertently be largely drank afterwards, even cold water, acidity of the whole mass may follow, resulting in a night of distress, if not actual or dangerous sickness. So it is better not to run the risk.

To derive a more decided medicinal effect, fruits should be largely eaten soon after rising in the morning, and about midway between breakfast and dinner.

An incalculable amount of sickness and suffering would be prevented every year if the whole class of desserts were swept from our tables during summer, and fresh, ripe, perfect fruits and berries were substituted, while the amount of money that would be saved thereby, at the New York prices of fruits, would in some families, amount to many dollars, dollars enough to educate an orphan child, or support a colporteur a whole year, in some regions of our country.

